# 1107. Case Study. Limb Salvage in a Patient with Extremity Burns Using Bioresorbable Antimicrobial Matrix over Dermal Matrix

**DOI:** 10.1093/jbcr/irag033.211

**Published:** 2026-04-06

**Authors:** Carmen E Flores

**Affiliations:** Leon S. Peters Burn Center, Las Vegas, NV

## Abstract

**Patient Presentation (age range, injury details, relevant history):**

We present a 74-year-old male with 15% TBSA fourth-degree contact burns to the bilateral lower extremities. His history included obesity and coronary artery disease, hepatitis C, and renal failure. During his hospitalization, his burns progressed to full thickness, severely threatening both lower limbs. Our institution was consulted six weeks after admission, at which point he had developed septic shock and multi-organ failure secondary to wound infection and osteomyelitis.

**Clinical Challenges:**

Limb salvage in patients with extensive lower extremity burns is particularly challenging due to high infection risk and limited options for durable coverage and functional restoration. Published data has demonstrated the potential for an antimicrobial bioresorbable matrix to enhance healing in diabetic patients with lower extremity ulcers at high risk for infection and limb loss. This case highlights the application of such a matrix with the primary goal of limb salvage in an elderly patient with complex burn injuries.

**Management Approach:**

Serial excisions were performed with excision and debridement of necrotic skin, tendon, muscle, and bone. Temporary allograft coverage was unsuccessful. Management included two separate applications of bovine collagen dermal substitute under the bioresorbable antimicrobial matrix, with adjunctive negative pressure wound therapy. During acute infection, daily wet-to-moist dressings with sodium hypochlorite solution were employed for a total of three days.

**Outcomes:**

Exposed muscle remained pale and nonviable with both limbs critically threatened following failed allograft coverage, repeat excisions, and complicated daily dressing changes. Skin substitute placement was attempted 25 days after the index excision. Within five days of the second application of the bioresorbable matrix over dermal matrix, both limbs demonstrated progressive vascularization and granulation tissue, suggesting salvageability. One month following matrix application, the patient was discharged to a skilled nursing facility with full coverage of tendon, muscle, and bone and a clean, well-granulating wound bed.

**Lessons Learned:**

Limb salvage was achieved through aggressive infection control, with the bioresorbable antimicrobial matrix playing a substantial role. Although allograft and other skin substitutes had been previously applied, no meaningful wound progression occurred until after matrix placement, demonstrating its potential to enhance healing in extensive, high-risk burn wounds.

**Applicability to Practice:**

This case supports the potential utility of bioresorbable antimicrobial matrices for limb salvage in extensive lower extremity burns refractory to conventional management.

